# Digital Interventions to Save Lives From the Opioid Crisis Prior and During the SARS COVID-19 Pandemic: A Scoping Review of Australian and Canadian Experiences

**DOI:** 10.3389/fpubh.2022.900733

**Published:** 2022-07-12

**Authors:** Andrea Donnell, Chandana Unnithan, Jessica Tyndall, Fahad Hanna

**Affiliations:** ^1^Program of Public Health, Department of Health, Torrens University Australia, Melbourne, VIC, Australia; ^2^Library & Learning Services, Torrens University Australia, Adelaide, SA, Australia

**Keywords:** opioid crisis, COVID-19, Australia, Canada, digital health (e-health), mobile apps (SaaS), public health, harm reduction

## Abstract

**Background:**

The potential for digital initiatives for opioid harm reduction is boundless. Synthesized evidence on current interventions and their efficacy are emerging. This scoping review is an effort to aggregate Canadian and Australian digital health initiatives used to prevent opioid-related deaths and minimize harm, prior to and particularly during the pandemic of SARs-COVID-19, when the crisis escalated.

**Methods:**

The Joanna Briggs Institute's methodological framework for conducting scoping reviews was used. Peer reviewed and gray literature published between January 2016 to October 2021 were included. Search translation was performed across CINAHL, Cochrane, SCOPUS, MEDLINE Complete, and ProQuest Public Health with consistent use of key search terms. Citation checks were also conducted. Studies included were written in English and reported on digital technologies to prevent opioid-related harm and/or mortality in participants aged 18 years or older in Australia and Canada.

**Results:**

A total of 16 publications were included in the final analysis (Australia = 5; Canada = 11). The most frequently reported digital technologies were telehealth to support access to treatment (*n* = 3) and mobile applications for overdose monitoring and prevention (*n* = 3). Telehealth-delivered opioid replacement therapy demonstrated equal outcomes and treatment retention rates compared to in-person and mobile applications for overdose monitoring demonstrated lifesaving capability through direct linkages with emergency response services.

**Conclusions:**

Digital interventions to minimize opioid crisis related harm and overdose prevention are fast emerging in Australia and Canada. During the pandemic, the crisis escalated in both countries as a public health emergency, and different initiatives were trialed. Digital harm reduction solutions via mobile apps (or SaaS solutions) were found to have the potential to prevent accidental overdose deaths and save lives, if rendered through privacy preserved, secure and trust enabled methods that empower users. Knowledge sharing between the two countries, relating to suitable interventions, may add significant value in combatting the escalating opioid crisis in the post pandemic era.

## Introduction

According to World Health Organization, more than 70% of the 500,000 deaths globally attributable to drug use are related to opioids (1). Globalization is playing a crucial role in the developing opioid epidemic, with high rates of opioid prescription, rising extra-medical (synthetic) opioid use, and diversification of the global opioid markets (including the proliferation of highly toxic synthetic opioids) highlighting the problem as a significant public health crisis. The differing socio-political and historical contexts of opioid crises across countries have undoubtedly influenced experiences and approaches to tackling the problem (1).

Canada is the second largest consumer of prescription opioids in the world, with over 20 million prescriptions for opioids being dispensed per year i.e., equivalent to nearly one prescription for every adult over the age of 18 years. In Australia, the National Drug Research Institute (NDRI) reported that extra-medical opioid use was responsible for 2,203 deaths and 32,000 hospital admissions during 2015–2016 (2). Equating to over 70,000 years of life lost, extra-medical opioid use is estimated to cost the Australian government $15.7 billion a year in both health and social costs. As a result of the escalating harm, Canada declared the developing opioid crisis a public health emergency in 2016, while Australia followed 3 years later in 2019 (2).

While the opioid crisis had been a declared public health emergency in Australia and Canada, during the pandemic period of 2020–2021, there was an escalation of the crisis. In Canada, the deaths by overdose in 2020 had exceeded COVID-19 related deaths, making it a twin crisis. Opioid harm reduction and treatment services faced considerable challenges in maintaining access for people that use drugs. These challenges include restrictions of in-person appointments, closure or reduced hours for needle and syringe programs, increased demand for drug treatment, and redeployment of health care staff to support COVID-19 responses (3–5). These challenges have been linked to increased levels of harm through sharing of injecting equipment and overdoses (3, 4). On the other hand, users of opioids had increased as a direct consequence to lockdowns, quarantines, and related mental health challenges (6). Reducing opioid-related harm measures to restrict supply, has resulted in a lateral substitutive shift in supply from one opioid category to the other (7).

Opioids are typically prescribed to manage pain often after a surgery or injury, or for certain health conditions. For example, Fentanyl is a synthetic opiate narcotic prescription drug used primarily for cancer patients with severe pain. Fentanyl is often added to illicit drugs such as heroin, cocaine, or methamphetamines as a powerful enhancer (2). The end user is often unaware of the potential danger and emergency response within the first 10 min of use is critical to the survival of people who have taken drugs contaminated/laced with *Fentanyl* (1).

The ongoing opioid public health crisis was further exacerbated by the COVID-19 pandemic (8). From the onset of the COVID-19 pandemic in Canada, 5,148 apparent opioid toxicity deaths occurred between April 2020 and December 2020, representing an 89% increase from the same period in 2019 (9). Similarly, in Australia, rates of self-reported non-fatal heroin overdose among those that regularly use stimulant drugs and regularly inject drugs are also escalating (10). A comparison of Australia and Canada was prudent due to similarity in the demographic profiles, healthcare systems and functions, and data coding for hospital and emergency presentations (11). Both countries have historically faced similar challenges in combating the opioid epidemic; however, the timeline differs as to when the crisis became a public health emergency.

Conversely, the ubiquitous availability of mobile technologies and the internet provide health care with new, innovative ways of working to complement traditional opioid use disorder (OUD) models of care by leveraging people's propensity to use digital devices (12). Digital health is the new umbrella term that encompasses e-health, m-health, health informatics, and integration of IoT devices (Internet of Things is the term used for the networking of devices embedded with sensors, software, and other technologies for connecting and exchanging data with other devices and systems over the internet) (13). A comprehensive understanding of how these technologies align with the strategic goals of both the Australian and Canadian opioid crisis-related public health strategies can provide insights on how digital initiatives can be used to address the current gaps. As studies are still emerging in the field, the objective of this research was to synthesize current/emerging initiatives, identify gaps and share key learnings to address this escalating public health emergency in both countries.

## Methods

A scoping review of the literature analyzing technologies available to reduce opioid-related harm was undertaken using the PCC framework and PRISMA-ScR protocol (14, 15). The wide range of technologies, methods, and results used in OUD research suggests that the use of a scoping review as described by Peters et al. (14) was the most appropriate methodology.

### Protocol Registration

The detailed protocol was registered on Open Science Framework DOI 10.17605/OSF.IO/QSDZ3 or https://osf.io/qsdz3/?view_only=2a09a035b2a6419aa7668e84a72606cb.

### Eligibility Criteria

Inclusion criteria were informed using the PCC guideline (16):

(P) Participants- Opioid users (medically and/or illicitly) aged 18 years and older in Australia and Canada.
(C) Concept-Digital health technologies to reduce opioid-related harm and mortality, including electronic technologies such as web or computer-based devices and m-health technologies such as mobile phones, tablet devices, and applications.
(C) Context- opioid-related harm reduction and overdose prevention.

### Database Search

Original articles in English were identified from a systematic search of five bibliographic databases including CINAHL, Cochrane, SCOPUS, MEDLINE Complete, and ProQuest Public Health. An eligibility publication year of 2016- was applied to the search criteria, to align with Canada's declaration of the developing opioid crisis as a public health emergency in 2016 (which Australia followed 3 years later in 2019) (2). All identified articles were transferred to Mendeley, a reference management software, to manage study records and all duplicates were removed.

### Search Strategy

The search strategy included a set of keywords based on the PCC inclusion criteria of opioid use, digital technologies, and harm reduction identified with the help of a library specialist. Studies were included if they were written in English and reported on digital technologies to prevent opioid-related harm and/or mortality in participants aged 18 years or older in Australia and Canada. The final search strategy for the databases is outlined in Supplementary Table 1.

### Information Sources

By applying the eligibility criteria, papers were screened for selection using titles and abstracts. Full-text papers were obtained and screened in their entirety for studies that could not be obviously excluded based on the information outlined in the *Title and Abstract*. The literature review was conducted by the first author and the results were validated by the third author. Studies that did not mention a specific digital intervention (e.g., scoping reviews of m-health applications in which the specific application is not named and described in detail) and those that did not use mortality or opioid-related harm in the context of addiction or overdose (e.g., HIV or hepatitis prevention) were excluded from the review. Systematic and other scoping reviews were excluded when they did not relate to Australia and Canada, however, reference lists of identified studies were reviewed to identify additional relevant studies and citation tracking was performed. The reviewer also contacted the authors of the primary studies, reviews, and subject matter experts for further information to complete the data extraction table (16).

### Data Extraction From Included Papers

When the relevant papers were selected, the following data were recorded in a spreadsheet: author(s), year of publication, context/setting, country, database source, aims/purpose, methodology, intervention type, intervention category, sample size, outcomes, and any key findings relevant to the scoping review question. As Australia did not have a specific opioid drug health strategy at the time of writing this paper, the digital interventions highlighted in this study are aligned to the Canadian opioid response: access to treatment; access to harm reduction; awareness and prevention; tainted drug supply (17).

### Study Quality Appraisal

In alignment with the JBI reviewer's manual, this scoping review did not assess the quality of the included studies (16). The goal of this review was to gain an overview of the digital health technologies used in relation to opioid use disorder to reduce harm and mortality, not to assess their technical quality although some relevant statistics are presented on their efficacies.

### Synthesizing Report Findings

The results of the scoping review were reported in alignment with the PRISMA-ScR Extension fillable checklist outlined in Supplementary Table 2. Data items were grouped according to types of digital intervention and presented in a tabulated format using the categories of intervention, citation, country, aims, methodology, and key findings, and subsequently aligned according to the core pillars of Australia's and Canada's Opioid Drug Health strategies.

## Results

### Selection of Sources of Evidence

The searches from the five electronic databases hit a total of 1,780 records [MEDLINE (*n* = 118), SCOPUS (*n* = 19), CINAHL (*n* = 20), ProQuest Public Health (*n* = 1,626), Cochrane Library (*n* = 7)] that led to a total of 1,699 published studies that were then screened after the removal of duplicates and texts before 2016 that had not been detected with the filters. The full search strategy for all databases and results are included in Supplementary Table 1.

**Figure 1:**
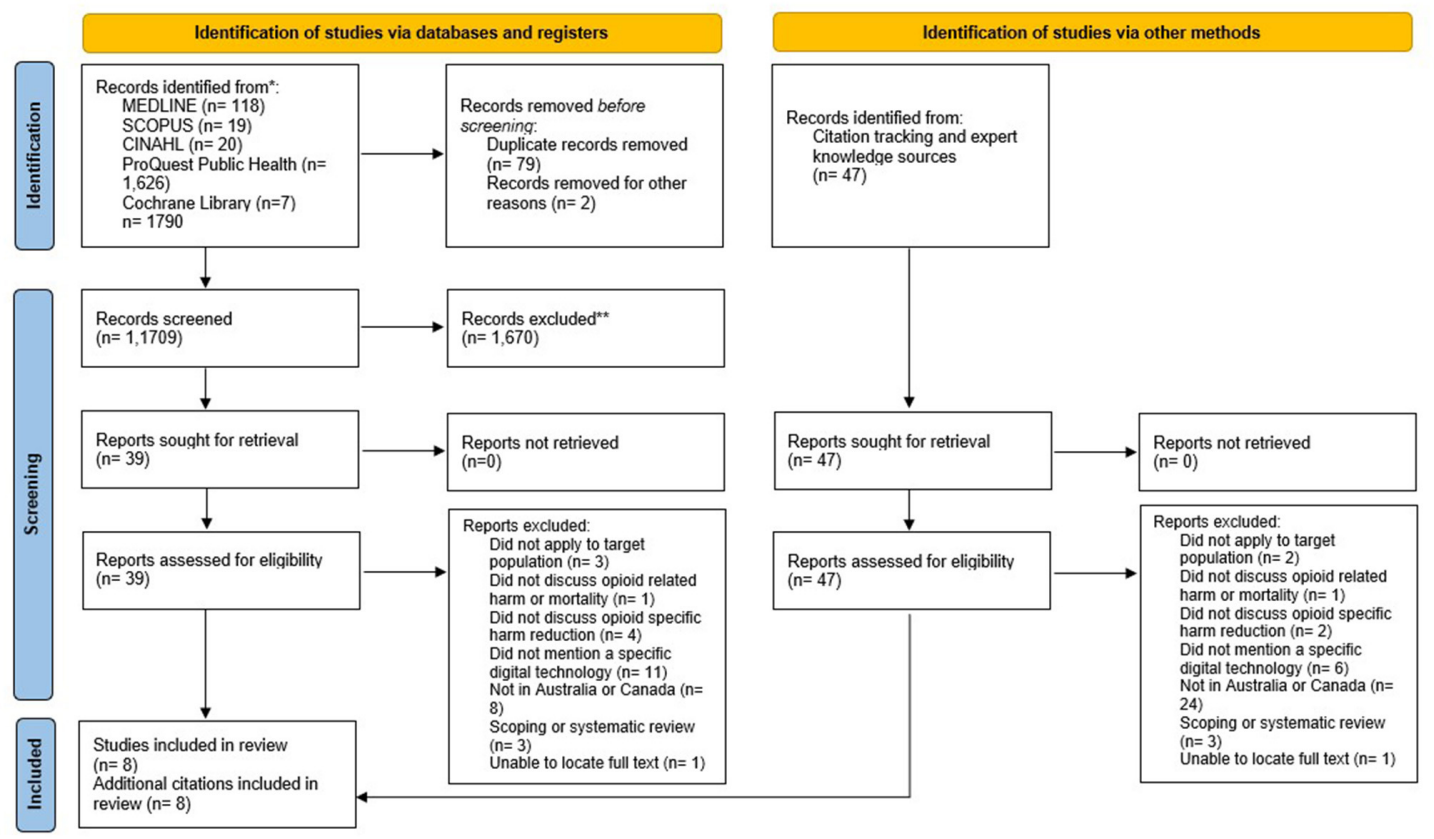
PRISMA-ScR 2020 flow chart of paper identification and selection process.

A total of 39 full-text papers were retrieved from the different libraries of which reference lists were checked identifying another potential 47 papers. The full-text screening stage of the potential 86 papers led to 70 being excluded for reasons outlined in the PRISMA flowchart in Figure 1. Therefore, a total of 16 papers were identified as being relevant to the scoping review and were included in the final data extraction and narrative account stage.

### Characteristics of Sources of Evidence

The research aimed to analyse and synthesize published data on digital interventions that aim to reduce opioid-related harm (mainly overdose death prevention) as well as related harm reduction services, within Australia and Canada. Sixteen papers were included in this review, 5 from Australia and 11 from Canada, and 50% (*n* = 8) described technologies related to harm reduction, 25% (*n* = 4) related to education and prevention, 19% (*n* = 3) related to access to treatment, and 6% (*n* = 1) related to reducing illicit supply pillars of the drug health strategies. The most frequently reported digital technologies were telehealth to support access to treatment (*n* = 3) and mobile applications for overdose monitoring and prevention (*n* = 3), followed by prescription dispensing monitoring programs (*n* = 2), overdose response buttons (*n* = 2), and drug checking (*n* = 2).

The reviewed studies have used three types of study design: 11 used quantitative methods, 1 used qualitative methods, and 2 used mixed methods. Two papers were government papers with no specified methodology. Among the quantitative studies, 7 were action research piloting new technologies, 2 were prospective cohort studies and 2 were retrospective cohort studies. The qualitative and mixed methods studies used a range of semi-structured qualitative interviews (*n* = 1), pre-post intervention surveys (*n* = 1), and intervention outcomes testing and surveying (*n* = 1).

## Findings

Access to treatment initiatives centers around the active treatment and management of substance use disorders with the goal of cessation or reduction of opioid use (17). Three studies explored digital technologies to support “Access to treatment” for OUD related to telemedicine (18–20). Eibl et al. (18) conducted a retrospective cohort study of patients initiating opioid agonist therapy (OAT) in the province of Ontario, Canada between 2011 and 2012 to understand the retention rates of patients treated *via* face-to-face vs. *via* telemedicine. Of the total 3,733 OAT initiating patients, those treated *via* telemedicine were more likely to be retained in therapy at 1 year than those treated in person at 50 vs. 39%, respectively (*n* = 1,590; aOR = 1.27; 95% CI 1.14–1.41; *p* < 0.001). Deacon et al. (19) trialed the use of telehealth to deliver the Australian Treatment Outcomes Profile (ATOP) which is a clinically validated tool used for clinical assessments, care planning, risk screening, and patient-reported outcome measurement in the alcohol and other drugs (AoD) treatment setting. Results found for all ATOP items, nearly all items (76%) reached moderate (0.7) or excellent (0.9) validity when administered over the phone when compared to face-to-face administration.

Morin et al. (20) conducted another retrospective cohort study of 55,924 patients enrolled in OAT in Ontario, Canada between 2011 and 2015 to assess the broader health outcomes of those receiving telemedicine delivered OAT compared to in-person. The findings of the above study reveal that when compared to in-person care, telemedicine was not associated with a higher rate opioid-related emergency department visits (IRR = 1.1, CI: 0.9–1.3) and hospitalisations (IRR = 0.9, 95% CI: 0.9–1.0), 1 year treatment retention (OR = 1.0, 95% CI: 0.9–1.1), or all-cause mortality (OR = 0.9, 95% CI: 0.8–1.0). However, patients who received predominantly telemedicine delivered OAT, had higher rates of overall emergency department presentations (IRR = 1.4, 95% CI: 1.4–1.5, 40% increase), mental health-related emergency department presentations (IRR = 1.5, 95% CI: 1.3–1.6, 50% increase) and mental health hospital admissions (IRR = 1.2, 95% CI: 1.1–1.3, 20% increase) (15).

Among the 4 papers aligned with the “education and prevention” (21–24), 2 were related to prescription dispensing monitoring programs (PDMPs) (21, 23), 1 was related to federal electronic medical records (22) and 1 was related to the digital collection of patients reported outcome measures (PROMs) (24). Of the two PDMPs, one was a federal government paper and did not list any results (21). The second paper evaluated the implementation of SafeScript—the only mandatory real-time PDMP in Australia which is used in Victoria (22). Using data collected through the Burnet Institute's SuperMIX study—a prospective cohort study of about 1,300 people who inject drugs-−20% (48/242) of participants had been refused a prescription of medication that is monitored by SafeScript. Of those that had been refused a script, 36% (16/44) were for the treatment of anxiety and 45% were refused two or more times by prescribers. Eight participants (3%) reported having a prescription for a medication that they were already using withdrawn (23).

The Australian Digital Health Agency (22) described the use of the federal “My Health Record” system that promotes the sharing of health information across state and territory jurisdictional boundaries to inform clinical decision-making. Although no conclusive information on opioid harm minimization is outlined, patient medication and prescription details are available on the system of which 23 million Australians currently use. Nielsen et al. (24) describe a protocol to test the implementation of computer-facilitated Routine Opioid Outcome Monitoring to deliver screening and brief interventions for opioid-related problems in community pharmacy practices in New South Wales and Victoria, Australia.

Access to harm reduction approaches is focused on reducing the level of harm associated with opioid use. The goal is not to promote the cessation of opioid use, but rather to help promote the use of substances in a safe and controlled manner to reduce the degree of harm and chance of overdose (17). The most commonly reported digital health interventions aligned with the “Harm reduction” drug health pillar were mobile applications (often known as Software as a Service or SaaS) for overdose monitoring and prevention (*n* = 3). The Lifeguard App (25) and BeBrave app (26) are designed to link people who use drugs (PWUD) (particularly alone) to emergency responders. The Lifeguard App was deployed by the Provincial Health Services Authority in BC, Canada in May 2020. As of August 2020, 1,700 people have used the Lifeguard App and recorded 5,200 uses and as of 23 June 2021, the app had saved 41 lives since being launched in the province of British Columbia. This solution is unique in its seamless connection to public health emergency service health responders through 9–1–1 services rendered by the provincial health services authorities and is the only endorsed service by 2 regional governments (in British Columbia and Northwestern Ontario). The BeBrave app reports that the service has received a total of 2,967 calls since 15 May 2020 and 33 rescue missions were undertaken (26). However, the mobile app connects with voluntary emergency response providers, who will then need to connect with 9–1–1 services and not direct with emergency services, which restricts its timely intervention capability. Bristowe et al. (27) outline a similar process to the BeBrave App, in which the user will outline the type of substance they plan on using and an operator will stay on the line checking in every 5–10 min. As this is a protocol, no results have been reported at the time of this paper.

The second most common digital health interventions in this category were the use of wireless overdose response button systems (*n* = 2), in which residents in supported accommodation (social housing) pushed a button before drug use to request staff to do a safety check on them. This intervention is based on the premise that opioid users are a specific disadvantaged population group residing in subsidized housing facilities. The BRAVE Technology Cooperative (28) reported 189 instances of safer use, 80 overdose reversals, and 160 instances of violence prevention. However, Bardwell et al. (29) conducted semi-structured qualitative interviews (*n* = 14) to examine the experience of women using this type of digital intervention and found participants were not using this solution as intended for their own drug use. Rather, it was being used for other emergencies such as gender and/or sex-work-related violence. Two papers that described using a combination of chemical analytical methods to provide drug checking services (30, 31). Mema et al. (30) describe drug checking at a major music festival in British Columbia, Canada, using fentanyl immunoassay strips and found that 1.6% (31/1,971) of samples returned a positive result for fentanyl. Those who tested for fentanyl were six times more likely to discard the sample on-site when faced with a positive result. Wallace et al. (31) describes a pilot program for a real time-multi-technology platform for drug checking that utilizes a suite of chemical analytical methods to provide harm-reduction advice in a community setting. As of July 2021, 2,213 samples have been tested of which 816 tested positive for fentanyl. Brave Technology Cooperative (32) describes a pilot program for the use of radar motion sensors in the restrooms of supported housing. As of the time of this paper, there had been no responses triggered.

Reducing illicit supply refers to initiatives that aim to disrupt the illegal and often tainted opioid supply chain (17). Tyndall (33) describes the use of an ATM that uses biometric scanning for people who use drugs to pick up their prescribed medications. No data regarding the uptake or usage was disclosed. The full results and key findings are outlined in Table 1.

**Table 1:** Characteristics of included studies.

**References**	**Context/setting**	**Aims/purpose**	**Methodology**	**Intervention type/description**	**Outcomes**	**Key findings that relate to the scoping review question**
**Access to treatment**						
Eibl et al. (18)	Canada Supervised clinical setting in Ontario	To evaluate the effectiveness of telehealth delivered OAT against traditional in person treatment by comparing treatment outcomes for both groups	Non-randomized retrospective cohort comparison	Treatment adherence and outcome monitoring Telehealth-delivered opioid agonist therapy	Patients treated *via* telemedicine were more likely to be retained in therapy than patients treated in-person (*n* = 1,590; aOR = 1.27; 95% CI 1.14–1.41, *p* < 0.001). Telemedicine patients demonstrated a retention rate of 50% at 1 year whereas in-person patients were retained at a rate of 39%. The mixed group also had a higher likelihood of retention than the in-person group (*n* = 418; aOR = 1.26; 95% CI 1.08–1.47; *p* = 0.001) and had a retention rate of 47% at 1 year	In addition to supporting specialist consults, the use of telemedicine can be expanded to facilitate the interaction of physicians and patients in a removed supervised clinical setting
Deacon et al. (19)	Australia Public sector specialist AoD treatment services in NSW	To validate the use of the Australian Treatment Outcomes Profile (ATOP) for administration over the telephone	Non-randomized prospective cohort comparison	Outcome monitoring Telehealth delivered Australian Treatment Outcomes Profile (ATOP)	107 AoD clients. Most ATOP items (76%) reached above 0.7 (good) or 0.9 (excellent) agreement between face to face and telephone use	Its validation for remote use over the telephone will support staff to monitor clients' risks and outcomes—of relevance in response to the COVID-19 pandemic in which services are increasingly relying on telework approaches to client monitoring When administered *via* the telephone, nearly all ATOP measures reached moderate or excellent validity compared to face-to-face administration
Morin et al. (20)	Canada Supervised clinical setting	To evaluate how telemedicine as a modality for opioid agonist treatment compares to in-person care	Retrospective cohort study. 55,924 individuals were included in the study	Treatment adherence and outcome monitoring Telehealth-delivered opioid agonist therapy	Receiving OAT by predominantly telemedicine was not associated with all-cause mortality (OR = 0.9, 95% CI: 0.8–1.0), 1 year treatment retention (OR = 1.0, 95% CI: 0.9–1.1), or opioid-related emergency department visits and hospitalizations when compared to in-person care. Patients that received predominantly telemedicine delivered OAT the rate of overall emergency department presentations (IRR = 1.4, 95% CI: 1.4–1.5, 40% increase), mental health-related emergency department presentations (IRR = 1.5, 95% CI: 1.3–1.6, 50% increase) and the rate of mental health hospital admissions (IRR = 1.2, 95% CI: 1.1–1.3, 20% increase) were all higher	Telemedicine is equal to in-person care regarding mortality opioid-related emergency department visits and retention and is a viable option for those seeking opioid agonist treatment
**Education and prevention**						
Australian Government Department of Health (21)	Australia National safe prescribing	Real time prescription monitoring program to identify patients at risk of dependence and medication harm	Government paper	PDMP An integrated digital platform that provides prescribers and dispensers with information about patient's history and use of controlled medicines to inform the prescribing or dispensing of medications	The NDE was developed and released in December 2018. Work with states and territories is continuing to integrate the NDE into their regulatory systems	Each state is responsible for developing its own PDMP prescribing and dispensing software. The national RTPM system is just the centralized reporting mechanism where state PDMP's such as Safe Script in Victoria or Dora in ACT and TAS feed into
Australian Digital Health Agency (22)	Australia Federal patient health record	To promote better patient and consumer outcomes through the sharing of health information to inform clinical treatment decisions	Government paper	Personally controlled electronic medical record (My Health Record) Mt Health record enables authorized health professionals to view shared health information across multiple treatment settings. Such as medical history, pathology and diagnostic reports and medicine and prescriptions	More than 23 million Australian's have a My Health Record. There are nearly 450M documents in the system uploaded by health care providers and consumers, of which 278M are related to the prescribing and dispensing of medicines	My Health Record is not mandated for patients and clinicians to use. As the system is patient controlled, they are able to deny a health service access to view their health information and also request that information not be shared to their My Health Record
Fetene et al. (23)	Australia Medication dispensing and prescribing monitoring in Victoria	To provide prescribers with access to a patient's prescription history for high-risk medicines to enable safer clinical decisions and reduce extra medical use	Prospective cohort study	PDMP Mandatory real time prescription monitoring program for high-risk medications to facilitate the early identification of treatment and support for patients who are developing signs of dependence	Data from the Burnet Institute's SuperMIX study found that 20% of people who were using a medication monitored by Safe Script have been refused a refill of their prescription and 3% have had a prescription for a medication they were already using withdrawn	SafeScript is the only mandatory real time PDMP in Australia Although Safe Script is intended to support reducing medication associated harms, the lack of integrated mental health and drug treatment services can exacerbate underlying conditions by ceasing medication treatments without appropriate supplementary services being available to support the transition
Nielsen et al. (24)	Australia Community pharmacy practices in New South Wales and Victoria	To test the implementation of computer-facilitated Routine Opioid Outcome Monitoring to deliver screening and brief interventions for opioid-related problems	Mixed methods. Pre-post intervention surveys	Outcome monitoring and opportunistic brief intervention Computer-facilitated outcome monitoring with inbuilt short based intervention (SBI) software that assesses opioid outcomes with domains aligned with a clinical framework	Protocol- not outcomes yet reported Participating pharmacies (*n* = 25 pharmacies) will each invite 20 patient-participants who are being prescribed opioids to be involved in the study (*n* = up to 500 participants in total)	A key barrier to successfully sustaining digital technologies is their failure to be integrated within existing clinical workflows
**Harm reduction**						
BRAVE Technology Cooperative (26)	Canada PWUD alone	To reduce the overdose risk for PWUD alone, by connecting them with someone who can assist in the event of an overdose	To reduce the overdose risk for People who use drugs (PWUD) alone, by connecting them with someone who can assist in the event of an overdose	Overdose monitoring and response Mobile application -“Be Safe”/Remotely supervised consumption Users will log onto the app before taking their drugs to connect with trained volunteer responders who is ready to assist in the event of an overdose	Total of 2,967 calls since May 15, 2020 Total rescue operations *n* = 33, calls escalated to 911 *n* = 5, calls escalated to emergency contact *n* = 2	Digital technology does not connect with emergency response services, instead signals volunteer first responders. This is not useful as these responders then would need to connect with emergency which delays the response The rationale for this app was to build trust with users who do not wish to be taken to the hospital or become known to police
Provincial Health Services Authority (25)	Canada Individuals who take opioid drugs	To reduce the overdose risk for PWUD (particularly those alone), by connecting them direct with emergency response services (9–1–1)	Action research	Overdose prevention and response The Lifeguard App deployed by the Provincial Health Services Authority (PHSA) *via* BC Emergency Health Services, as the only endorsed government (public health) service It requires that PWUD pushes a timer button before taking a drug. The app will sound an alarm after a pre-specified timeframe has elapsed. If the PWUD has not canceled or extended the timer, the alarm begins, gets louder simultaneously sending a message direct to 9–1–1 services in the province which will dispatch paramedics immediately	As of September 2021, 6,649 people had used the Lifeguard App and 68,356 recorded uses. Out of these, an alert was sent 101 times to the British Columbia Emergency Health Services para medic dispatchers' team 72 people were able to speak to emergency dispatchers and confirm that they were ok, and 23 people required paramedics to be called to the scene for emergency medical attention	At the end of September 2021, the app has saved 40 lives since being launched in the province of British Columbia, and is the only service authorized by the regional government (Ministry of Mental Health and Addictions) and public health authorities that connects seamless/direct with 9–1–1 emergency response services
BRAVE Technology Cooperative (32)	Canada Supported housing restrooms (SRO rest rooms)	To prompt a safety check on people that have been in the bathroom for a prolonged period	Action research	Overdose monitoring and response Movement detection sensor (Brave Sensors). Uses radar, and motion sensors to detect a moving person, if they become still in a washroom. If it detects stillness or if the person has been in the washroom predefined as “too long” the system alerts by sending a text message to the designated responder phone to prompt a safety check	No responses triggered yet	N/A
**Harm reduction continued**						
Bardwell et al. (29)	Canada Supportive housing (Single Room Occupancy residences or SROs)	To examine the experiences of women in a supportive housing environment using a wireless overdose response button system	Semi-structured qualitative interviews	Overdose monitoring and response Wireless overdose response button system. Allows PWUD alone in their room to press a wall-mounted battery-powered button (about 2.5 cm in diameter) before their drug use, which sends a notification to a cellular phone monitored by building support staff who will check on residents and respond as required	Most participants indicated that they did not regularly press the button for their own drug use They spoke about utilizing the button in response to other people' overdosing, whether in their rooms or elsewhere in the building, detracting the app use from themselves or in response to physical violence and safety issues	Technological interventions need to take into consideration the structural vulnerabilities of the various sub-populations of PWUD Recommended co-design with the intended users to ensure that the intervention meets their actual needs to reduce harm from opioid crisis and reverse overdose deaths
BRAVE Technology Cooperative (28)	Canada Supportive housing environment (SROs)	To reduce the overdose risk for PWUD (particularly those alone), in supported housing by connecting them with a staff member that can do a safety check	Action research	Overdose monitoring and response Wireless overdose response button system (Brave Buttons). Blue tooth buttons are connected to a chatbot that when pressed triggers a text message to a designated phone number to request for assistance in supported housing environments	189 instances of safer use; 80 overdose reversals 160 instances of violence prevention	Does not just support safe opioid usage, rather is used as a physical safety mechanism Intended purpose of the intervention is not fulfilled in the opioid crisis harm reduction
Mema et al. (30)	Canada Recreational drug use	To describe participation in, and results of, drug checking (including fentanyl screening by immunoassay strips) at Shambhala festival and disposal of contaminated substances	Mixed methods. Drug testing with qualitative surveying	Drug checking using fentanyl immunoassay, strips, in which results along with safety tips were posted in real-time on an electronic screen for participants to read and decide if they wanted to safely discard them or not	1,971 samples were tested for fentanyl using immunoassay strips, of which 31 (1.6%) tested positive. Among samples tested for fentanyl, 51 (2.6%) of the 1,940 negative samples and 5 (16.1%) of the 31 positive samples were discarded on-site at an amnesty bin in the ANKORS tent. Those who tested for fentanyl were six times more likely (16.1 vs. 2.6%) to discard the sample on-site when faced with a positive result	Participation in fentanyl drug checking was high among festival guests (traditionally recreational users of illicit stimulants), none of whom expected their substance to contain an opioid Findings suggest that drug checking may trigger a behavior change, possibly by creating the space for a 'teachable moment' at a time when clients are most receptive
**Harm reduction continued**						
Wallace et al. (31)	Canada Community drug checking service	Pilot project utilizing a multi-technology platform utilizing a suite of instruments for point-of-contact drug checking as a harm reduction service integrated within community sites. treatment outcomes for both groups	Action research	Drug checking Uses several types of drug checking technologies (ATR-IR absorption, Raman scattering, Surface-enhance Raman scattering, gas chromatography coupled with mass spectrometry and test strips) to test illicit drug samples. Results are collated and reported in real-time using a web application and results are communicated back to the client either verbally, written, or *via* an online portal	As of the 30th July 2020, 2,213 samples have been tested of which 816 tested positive for fentanyl	Drug checking services can be delivered with minimal face to face contact to adhere to public health measures in response to COVID-19
Bristowe et al. (27)	Canada Community setting (protocol)	The primary objective of the study is to establish the feasibility of a virtual overdose response service with people with lived experience operators	Action research (pilot project)	Outcome monitoring Virtual (telephone-based) overdose Response The user will call a hotline and outline what type of substance they plan on using and the operator will stay on the line checking in every 5–10 min. If the participant doesn't respond when the operator emergency services are contacted. If they do respond every time after 30minutes the call is disconnected	Protocol- no outcomes yet reported	N/A.
**Reducing illicit supply**						
Tyndall (33)	Canada Opioid dispensing in Vancouver	To provide safe access to medical grade hydromorphone to reduce accidental opioid overdose through tainted illicit supply	Action research	Safe supply An ATM that uses biometric scanning for people who use drugs to pick up their prescribed medications The scanners read the vein patterns in a person's palm as an identifier, delivering and tracking their prescription in real-time	None specified	Advantages include reduced stigma and autonomy, 24/7 access, individualized dispensing programs that suit the needs of the person (daily, weekly, and monthly), secure real-time reporting and monitoring to trigger wellbeing and follow up check-ins, cost-effective as requires minimal staff to operationalise and does not require person-to-person contact which is an important public health measure during COVID times

## Discussion

The issues associated with problematic synthetic opioid use are linked with historical prescribing practices that have coincided with changes in drug use patterns over a long period, which culminates in a crisis (34). For example, an increase in opioid prescribing and utilization followed by rises in heroin use has led to the current widespread fentanyl availability and utilization in Canada. In response to the alarming number of opioid related deaths and dependence, a number of well-intended supply reduction initiatives such as changes to pain management and prescribing guidelines, prescription drug monitoring programs, regulation and up-scheduling of opioid medications, and changes to drug formulations to make them tamper resistant and difficult to use intravenously, resulted in large numbers of people that were opioid dependent unable to obtain prescription opioids (7). The displacement of pharmaceutical opioids without appropriate measures to support the demand side, drove both medical and non-medical opioid users to seek more readily available alternatives, evidenced by a sharp increase in heroin use in Canada between 2010 and 2015 and also heroin related overdoses (7, 34) This phenomenon left large sub-groups of opioids users with a drastically shrinking supply of pharmaceutical opioids. Declining supply with continued demand also drove up market prices, which coupled with socio-economic disadvantage shifted the supply routes to the illicit market (7, 35). Reducing opioid-related harm measures to restrict supply resulted in a lateral substitutive shift in supply from one opioid category to the other (7).

Unlike heroin, which is derived from the poppy plant, synthetic opioids, such as *Fentany*l can be manufactured anywhere, and manufacturers are much more easily able to import the required chemicals to produce fentanyl due to poorly regulated global distribution systems. *Fentanyl* is cheaper to purchase and manufacture, easier to obtain (*via* crypto markets and *via* illicit pharmacies online) and the higher potency means suppliers source it in smaller quantities, making it easier to smuggle across international borders to maximize profit and reduce profit loss if intercepted by authorities (35, 36).

Australia's experience with extra-medical opioid use differs from the Canadian experience in the patterns of drug use, trends, illicit markets, and strong border controls. Due to the lower cost of prescription opioids, heroin, and methamphetamine in Australia, this is not a driver for the need to source cheaper alternatives such as Fentanyl, as is in Canada (37, 38). Although prescription opioid use in Australia has increased substantially between 1990 and 2018, the rates are still lower than in Canada. Regardless, there remain serious concerns that Australia is mirroring the same pathway that has contributed to the opioid crisis in Canada. The number of unintentional drug-induced deaths involving opioids has nearly tripled in the last 14 years with the crisis being primarily driven by pharmaceutical prescribing (39). Simultaneously, since 2012 the number of deaths involving heroin has also increased by over 65% (40). Comparatively in Canada, the primary drivers of the opioid crisis have shifted over time from over-prescribing to more toxic synthetic opioids (fentanyl) entering the illicit supply chain (41). As of 2019, the escalating opioid crisis and associated harms were already calling for innovative strategies in both countries.

The findings of this scoping review reveal innovative digital health initiatives for opioid crisis prevention that are emerging in Canada and Australia where it has been declared as a public health emergency which has been exacerbated throughout the SARS COVID-19 pandemic.

### Access to Treatment

#### Telehealth

COVID-19 has rapidly accelerated the implementation of telehealth in Australia, necessitated by socially distanced health care to protect both health care workers and patients. This has created a unique opportunity to leverage telehealth into routine clinical practice for OUD. The use of telehealth to support people with OUD was recently validated by Deacon et al. (19). Telephone support was identified as superior for this cohort as they do not have to rely on an internet connection and literacy levels as is a requirement for some other remote monitoring initiatives.

Opioid agonist therapy (OAT) is a harm reduction model of care where opioid agonists such as buprenorphine/naloxone or methadone, are substituted to replace more dangerous and addictive opioids to stabilize a person's use and maintain an individual's psychosocial functioning (18). Patients often remain in therapy for several years and are more classified as a maintenance treatment, in which the person will slowly reduce the dosage to safely wean off the medication, by alleviating the opioid withdrawal symptoms (20).

The rigidity of traditional treatment paradigms for opioid use disorder characterized by frequent clinic appointments, supervised dosing, screening, and limited take-home doses of medications poses significant challenges for easily incorporating it into everyday life. The flexibility of telehealth to support opioid agonist therapy adherence offers an effective alternative to in-person care with people 50% more likely to be retained in therapy for 1 year compared to 39% for those treated in person (18). These results support findings from other studies, demonstrating that telemedicine-delivered OAT is a comparable treatment modality with the potential to expand access to treatment for those with opioid use disorder and may be an effective and appropriate alternative to traditional face-to-face treatment modalities (18, 20, 42).

Despite telehealth offering greater flexibility and potential access to services, there are challenges with supporting telehealth engagement among marginalized patients. Telehealth-only models of care have been associated with increased rates of mental health emergency department presentations and hospitalisations (20) highlighting the need for further research to understand the mental health implications of telehealth use for those undertaking OAT. Additionally, the lack of standardized clinical guidance, funding and resource constraints, jurisdictional boundaries and lack of collaboration between governments, continue to pose significant challenges to the successful implementation of holistic telehealth care for PWUD in Australia (43–46).

### Education and Prevention

#### Prescription Dispensing Monitoring Programs

The use of PDMPs is a core feature of both the Canadian and Australian opioid management strategy and supports safe and accurate prescribing of high-risk and controlled substances following best-practice guidelines to reduce polypharmacy and patient harm (47). PDMPs are available in seven of ten provinces in Canada (36), while in Australia only Victoria (one of eight jurisdictions) has real-time script monitoring (34).

The Australian Government Department of Health (21) describes the rollout of national real-time prescription monitoring (RTPM). Devolved governance means each jurisdiction is responsible for developing/procuring its own PDMP prescribing and dispensing software, resulting in multiple medication systems that are difficult to integrate. With the expansion of electronic medical records and the national My Health Record system (22) there is potential to enable prescription monitoring in some capacity.

My Health Record is a personally controlled universal electronic health record summary that is linked with an Australian resident's Medicare number. The rationale for the introduction of the My Health Record in Australia is to provide a centralized health record for patients to improve the coordination of care, reduce duplication and medication harm, and enhance patient control of their health information to promote shared decision making (22). The system allows individuals and their treating team to upload clinical documents, pathology and diagnostic reports, medication and immunization histories to a secure online record that is controlled by the patient. Only permitted health care providers can access an individual's My Health Record and patients also have the ability to restrict certain information being viewed in their record (22). Therefore, despite the My Health Record System having the future capability for prescription monitoring, the challenge therein lies with the ability of patients to remove information from their health record, such as opioid prescriptions, that works counter intuitively to efforts to reduce users from acquiring medications from clinicians in different states.

SafeScript became mandatory in Victoria in April 2020, making it the only mandatory real-time PDMP in Australia (23). The use of PDMPs globally is well-documented (34, 48–50), however, the evidence of their effectiveness to reduce opioid-related harm and consequences is mixed. Fente et al. (23) explored the effects of SafeScript in Victoria, interviewing 387 people. Since the introduction of SafeScript in April 2019 they found that 20% of participants had been refused a prescription medication that is monitored by Safe Script. Of those that had been refused a script, 36% were for the treatment of anxiety and 45% were refused two or more times by prescribers. Alarmingly, 3% reported having a prescription withdrawn for a medication that they were already using.

A high number of people use medications extra-medically and have comorbid mental health disorders that require multidisciplinary support. Although SafeScript is intended to support reducing medication-associated harms, the lack of integrated mental health and drug treatment services can exacerbate underlying conditions by ceasing medication treatments without appropriate supplementary services being available to support the transition.

The correlation of increased opioid-related mortality following restrictions of prescribed medications is well-documented (34, 47, 51, 52), which highlights the need for ongoing research to understand the impact of RTPM use in Australia and opportunities to better integrate digital health infrastructure to support people who use drugs (23).

A systematic review conducted by Rhodes et al. (47) found that as a standalone feature, PDMPs were ineffective in reducing several indicators of population-level opioid-related harm such as opioid dependence, hospital separations, emergency department utilization, and usage levels of both pharmaceutical and illicit opioid use. Limitations of PDMPs, however, do not necessarily lie with the technology itself but rather the ineffective use of the information generated to inform clinical decision-making. This highlights the need for further formally documented research on the use of PDMPs to combat the opioid crisis and their effectiveness across various populations in Australia to ensure that the results are generalisable.

### Harm Reduction

#### Overdose Response and Monitoring

BRAVE Technology Cooperative (26) and the Provincial Health Services Authority (25) describe the use of mobile apps that link people who use drugs alone to emergency responders, to reverse an overdose. The premise is based on prioritizing the autonomy, anonymity, and privacy of those that use the service to allow them to decide the appropriate emergency response for them in the event of an overdose. Accordingly, BeSafe is a mobile app where the person specifies their location, the drug they are using, whether they have naloxone, and their emergency response plan. This is completed before the person uses drugs. Through the app, the person can call the BeSafe Support Center. The pre-supplied information is hidden from the call supporter unless the operator suspects the caller is overdosing. In the event of a suspected overdose, the caller will be given a 20 s countdown alert advising their information will become available unless they dismiss the alert to indicate that they are ok. If the alert is not dismissed, the caller's location and emergency response plan will be shared with the supporter who uses it to send for help, either someone specified by the caller or emergency services (26). However, the app does not connect directly to emergency health responders as it is operated by a co-op. It also relies on a call center that is operated by voluntary responders who will use their judgement of a suspected overdose.

Conversely, The Lifeguard App was deployed by the Provincial Health Services Authorities via the government emergency services in a provincial health intervention model, in Canada. It combines GPS and geofencing technologies to connect the user to emergency services, in case of an emergency. It requires that the PWUD push a timer in the app before they take any drug. There is facility to extend the timer. If the timer is not canceled within 45 s, an alarm begins, and the emergency paramedic services (in BC, 9–1–1 services) are called. As all information is deleted from the app after a call is linked to the paramedic services, there is privacy preservation and anonymity assurance that has led to the increased uptake of this mobile app (or SaaS digital intervention).

Since May 2020, these 2 apps have had a combined 8,167 calls and saved 66 people from overdoses (26, 53). Despite the success in saving lives, qualitative studies to understand the acceptance of digital health interventions are still emerging. Concerns about security, privacy, and accessibility remain an important consideration, while public health authorities are considering the implementation of novel digital protocols such as blockchain (54). Supporting substance users wish to remain anonymous is essential to avoid the stigma of being identified by family, employers, or law-enforcement authorities. Therefore, they hesitate to use any digital intervention such as the Lifeguard App which may accidentally reveal their identity if a hacker intercepts an emergency call. To mitigate this, public health authorities are considering the development of end-to-end secure transmission methods where a call for reaching emergency services cannot be intercepted as it would be fortified by a layer of blockchain or distributed ledger technologies (DLTs). By working within this secure ecosystem, the users can be assured of their privacy. In addition, permissioned blockchain protocols can be configured with artificial intelligence methods such that users can get support from a pre-approved peer group while remaining anonymous.

Although mobile phones have penetrated 90% of the global market, people with transient lifestyles, and uncertain living and employment arrangements may not have consistent access to mobile technologies, Wi-Fi, or safe spaces to comfortably use the digital health interventions, which impacts the accessibility of these innovations (4, 5, 55). Thorough needs assessments that take into consideration these logistic and functional limitations as well the dangers of drug use, user preferences, interpersonal relationships with friends, and socioeconomic status are necessary to not further divide already marginalized populations.

A pilot of a Wireless overdose response button system, in which people in supported residential accommodation press a wall-mounted battery-powered button before their drug use, triggering a notification to a cellular phone monitored by building support staff who check on them and respond accordingly (28) was reported. The findings show 189 instances of safer use, 80 overdose reversals, and 160 instances of violence prevention. Semi-qualitative interviews to understand the experiences of women using this type of digital technology within a supportive housing environment found that participants did not use the overdose prevention button as intended (using it before individual drug use) rather it was mostly reported to be used for other emergencies such as other residents or guests overdoses and sex-work related violence. This demonstrates that technological interventions for OUD need to be co-designed with intended users and take into consideration the inherent vulnerabilities of sub-populations within this cohort. PWUD need to be empowered to use their agency to assess their levels of perceived risk. This cohort of women did not perceive their overdose risk as high as those around them, demonstrating a unique learning opportunity to tailor interventions to meet the specific needs of women who use drugs alone to avoid gender-based violence (29).

The Brave Technology Cooperative (32) describe the use of passive surveillance in bathrooms in supported accommodation as a means of overdose detection. The Brave sensor uses radar, which uses motion sensors to detect a moving person if they become still in a washroom. If it detects stillness or if the person has been in the washroom predefined as 'too long', the system alerts by sending a text message to the designated responder's phone to prompt a safety check. Despite the success of passive surveillance sensors in the US, the BraveSensor trial in Canada as of July 2021 has yet to trigger any responses (32). Although the potential of this type of digital health intervention is well-understood key ethical and implementation considerations such as liability, data privacy and security, technical accuracy, consent, communication, and stigma pose challenges to the scalability of this type of digital health intervention (56).

#### Drug Checking

Mema et al. (30) describe drug checking at a major music festival in British Columbia using fentanyl immunoassay strips to detect fentanyl in recreational substances. The challenge with deploying mobile technologies for drug-checking is that it is difficult to ensure consistent, reliable results that can be easily interpreted without specialized training (57). Test strips that are simple to use are essential to frontline harm-reduction settings, however, they cannot provide information beyond a binary yes/no result (31). This makes it difficult for people to make truly informed decisions about their level of risk as they have no information about how potent the sample is. Although these interventions do not deter long-term drug-taking, it does empower people to make informed decisions based on risk when fentanyl is detected by providing information on potency (58).

In the pilot protocol outlined by Wallace et al. (31) utilizing multi-model methods is discussed for disseminating drug-checking results, either in-person or through a web-based portal. The rationale for the use of multiple technologies is that the strengths and limitations of each technology complement each other, providing more comprehensive results. These findings are supported by Tupper et al. (58) who conducted a drug checking feasibility study using Fourier transform infrared (FTIR) spectrometer and fentanyl immunoassay strips in Vancouver and found out of 907 samples of heroin tested, 90.6% (822) tested positive for fentanyl. This not only highlighted the prevalence in the Vancouver drug supply but the acceptability and necessity of this type of digital health intervention as a harm-minimization and risk deterrent. Although these interventions do not deter long-term drug-taking, it does empower people to make informed decisions based on risk when fentanyl is detected by providing information on potency (59). Findings suggest that drug checking may trigger a change in behavior, possibly by creating the space for a 'teachable moment' at a time when clients are most receptive (30).

### Reducing Illicit Supply

As presented in the findings, Tyndall (33) had highlighted MySafe—a digital health intervention piloted in Vancouver, Canada that functions as an ATM with biometric scanning allowing PWUDs to collect prescriptions and offers real-time tracking of collection. There is tremendous potential to reduce illicit drug supply using this intervention. The reduced stigma, 24/7 access, customized dispension programs, secure real time tracking and reporting, follow-up checks, cost effectiveness with minimal staff requirement to operationalize this contactless solution—are all features that have the potential for scalability to other jurisdictions in Canada and globally. Nonetheless, the lack of formal evidence-based evaluation in academic literature thus far, is a limitation that can be addressed.

## Limitations

This review applied a systematic and rigorous search strategy but was limited in sample size, although the review indicates the potential of digital technologies to address the challenges of the Australian and Canadian opioid crises and the current gaps in approaches to utilizing them effectively. Despite efforts to comprehensively search literature, it is possible that some relevant papers were not included due to search terms or database restrictions. Additionally, some relevant papers may have been inadvertently omitted as this research did not focus on digital health-specific literature that is outside the scope of the public health domain, thus offering differing perspectives.

Furthermore, this review may have limited identification of all the digital technologies and SaaS/SaMD interventions that have been developed in response to the changing needs of opioid users during the COVID-19 pandemic due to publishing delays and challenges with timely dissemination of results from pilot projects. As this topic is evolving, it is recommended that the findings of this review be confirmed with a systematic review when more data is available.

## Call-To-Action

A key challenge with digital health is its dynamic nature. Delays in the dissemination of findings are proving to be a barrier to the scalability and sustainability of such technologies, meaning that these interventions are occurring in isolation, resulting in variations in clinical practice for the management of opioid use disorder.

This review has highlighted the lack of longitudinal studies to understand the long-term impacts of harm-reduction interventional strategies and robust mechanisms and evaluations of digital health implications that target both those that use opioids and healthcare/support workers within the two countries, where the universal health care system is the foundation. The different data sources in this review also produced varying results that limits the ability to quantify the extent of extra-medical use of opioids and associated harm and making it difficult to fathom the extent of the problem and how to best address the crisis.

This study reveals a need for more publications to demonstrate the potential beneficial impacts of digital health interventions to reduce opioid-related harm and mortality. Such studies will assist in developing a best practice foundation for implementing digital health interventions to reduce opioid-related harm across the various subpopulations. In Australia, digital health has become mainstream over 2020–2021, and during the same time, in Canada, digital technologies have been rapidly deployed as harm reduction tools. While the crisis is at different levels in the two countries, some of the initiatives and key learning can be shared across the two countries. This research is an invitation to the public health sector and policy makers to explore such innovations for reducing the harm caused by the opioid crisis.

## Conclusion

This review highlights there are gaps in Australia and Canada's approaches in managing their respective opioid crises. The COVID-19 pandemic has created the opportunity to incorporate digital health initiatives into the core strategy of opioid public health measures, however, the lack of credible research and investment in digital interventions means that we are still yet to see its full potential in this space. The digital health interventions outlined in this review including telehealth-delivered opioid replacement therapy, drug checking, digital information sharing have resulted in behavioral changes in drug users. Conversely, Lifeguard App (a mobile app that directly connects to emergency services) has already saved more than 40 lives during the pandemic (53). As we shift into the era of “COVID normal,” the era of socially distanced health care will continue, impacting how people interact with each other and the health care system. There is a need to establish an evidence base that compares the Australian opioid public health crisis with the Canadian context to obtain key learnings that can be shared to prevent opioid-related harm and mortality.

## Data Availability Statement

The original contributions presented in the study are included in the article/Supplementary Material, further inquiries can be directed to the corresponding author.

## Author Contributions

CU and AD: conceptualization and visualization. AD and JT: methodology. AD: formal analysis, investigation, and writing—original draft. AD, CU, and JT: resources. AD, CU, and FH: writing—review and editing. CU: supervision. All authors contributed to the article and approved the submitted version.

## Conflict of Interest

The authors declare that the research was conducted in the absence of any commercial or financial relationships that could be construed as a potential conflict of interest.

## Publisher's Note

All claims expressed in this article are solely those of the authors and do not necessarily represent those of their affiliated organizations, or those of the publisher, the editors and the reviewers. Any product that may be evaluated in this article, or claim that may be made by its manufacturer, is not guaranteed or endorsed by the publisher.
