# Supplementary material for: Digital Interventions to Save Lives From the Opioid Crisis Prior and During the SARS COVID-19 Pandemic: A Scoping Review of Australian and Canadian Experiences
Source: Front Public Health. 2022 Jul 12;10:900733. doi: 10.3389/fpubh.2022.900733 (PMC9314644; doi:10.3389/fpubh.2022.900733)
Supplement: Supplementary file 1 [file Table_1.pdf]

Supplementary Table 1: Summary of search strategy.

**Serial #**	**Descriptions**	**Search terms**	**MEDLINE Complete**	**SCOPUS**	**CINAHL**	**ProQuest Public Health**	**Cochrane Library**
**1.**	**Population-opioid users**	("opioid overdose" OR "opioid misuse" OR "opioid use disorder" OR "People who inject drugs" OR PWID OR "opioid user*" OR "extra-medical opioid use" OR heroin OR opioids OR fentanyl)	170,361	215,219	215,219	215,219	37,924
**2.**	Concept	("digital health" OR mHealth OR "mobile health" OR e-health OR computer OR "mobile app*" OR "Mobile phone" OR smartphone OR "mobile device" OR telemedicine OR psychometric* OR "wearable sensor" OR technolog*)	2,719,726	8,403,575	395,471	1,219,595	99,268
**3.**	Context	(“ Harm minimis*” OR “ harm reduc*” OR mortality OR prevent* OR hospitali*)	4,113,880	4,621,859	1,201,108	1,866,317	340,846
**4.**	Location	(Australia* OR Canad*)	N/A	N/A	N/A	N/A	N/A
**5.**	Language	English	N/A	N/A	N/A	N/A	N/A
**6.**	Timeframe	01/01/2016 -01/10/2021	N/A	N/A	N/A	N/A	N/A
**7.**	1 & 2 & 3	Population & Concept & Context	1,370	1,362	426	10,673	375
**8.**	1 & 2 & 3 & 4	Population & Concept & Context & Location	181	51	**20**	4,138	14
**9.**	1 & 2 & 3 & 4 & 5	Population & Concept & Context & Location & English	181	51	20	4,130	14
**10.**	1 & 2 & 3 & 4 & 5 & 6	Population & Concept & Context & Location & English & Timeframe	118	19	20	1,626	7
